# Evaluating the impact of housing market liberalization on the timing of marriage: Evidence from Egypt

**DOI:** 10.1080/00324728.2021.1914853

**Published:** 2021-05-06

**Authors:** Ragui Assaad, Caroline Krafft, Dominique J. Rolando

**Affiliations:** 1 University of Minnesota,; 2 St. Catherine University

**Keywords:** marriage, housing, house prices, rent control, market liberalization, living arrangements, Egypt

## Abstract

The transition to adulthood around the world is increasingly characterized by young people’s desire to form independent households. Forming such households in Egypt generally requires buying or building a dwelling or obtaining a rental unit. Policies governing housing markets, such as rent control, and limited financing options have historically made access to housing for young couples challenging. In this paper, we use a difference-in-difference approach to evaluate how the liberalization of rental markets in Egypt affected the timing of marriage. We find that Egypt’s 1996 rental reforms accelerated marriages and led to a reversal in the trend of rising age at marriage.

## Introduction

Many governments around the world pursue housing policies that attempt to make rental housing more affordable, but such policies may lead to reduced supply of rental units (McCall 1988a, 1988b; Early 2000). Rent control policies in particular are intended to limit rent increases and protect tenants, but such policies also limit the housing available to new entrants to the housing market. Given the increasing global trend towards independent living at marriage (Bongaarts 2001; Lloyd 2005), housing markets can strongly interact with family formation.

This paper uses the case of Egypt—which liberalized its long-standing rent control laws in 1996—as a natural experiment to evaluate the effect of rent liberalization on the timing of marriage. In the 1960s, the Egyptian government imposed strict rent controls, with rents fixed in nominal terms at the start of rental contracts. Contracts were, by law, of indefinite duration, and could even be passed across generations through inheritance. In 1996, the Egyptian government introduced the ‘new rent’ law, which allowed new rental contracts (signed after the adoption of the law) to be of limited duration and gave landlords the flexibility to adjust the rent on renewal (Assaad and Ramadan 2008; Middle East Library for Economic Services 2011; Shawkat 2020). Previous work has suggested that the reversal in the rising trend in the age at marriage in Egypt may be attributable to the adoption of these housing market reforms (Assaad and Ramadan 2008; Assaad and Krafft 2015a). However, these studies did not undertake a detailed and rigorous policy evaluation. We use a difference-in-difference method to determine the causal impact of these reforms on the timing of marriage in Egypt.

In addition to the natural experiment of rent liberalization, what makes the Egyptian case particularly interesting for studying the relationship between housing markets and family formation is the prominent role that housing plays in marriage negotiations. Marriage in Egypt is essentially a contract between families, where the groom’s side is expected to provide housing (Hoodfar 1997; El Feki 2013). With increased expectations of independent living at marriage, housing availability for newly married couples takes on increasing importance.

The global literature on rent control has identified a number of different impacts of such policies on the price, quantity, quality, and distribution of housing. Rent control benefits the tenants of controlled units, but reduces supply overall and increases the prices of uncontrolled units, which yields negative average benefits and increases inequality (Early 2000; Roland and Svarer 2002; Diamond et al. 2018; Mense et al. 2018). The quantity of rental units decreases as a result of rent control, as does the quality (Gyourko and Linneman 1990; Sims 2007; Diamond et al. 2018). The design of rent control policies (particularly their rigidity) affects their impacts (Zheng et al. 2007). Studies have shown that rent control can affect other economic outcomes beyond (but related to) the housing market, for instance causing longer unemployment durations, reduced labour mobility, longer commute times, or increased religious conflict (Krol and Svorny 2005; Svarer et al. 2005; Field et al. 2008).

Our paper is the first, to our knowledge, to research the impact of rent control on family formation. Housing affordability, in general, is an important determinant of household formation outcomes around the world (Franc 2006; Farzanegan and Gholipour 2016; Di Stefano 2019; Xu et al. 2019; Su et al. 2020). While this paper pertains to Egypt, with its specific patterns of family formation and distinctive housing regulations, it could be relevant to other contexts where family formation is increasingly tied to independent living arrangements and where restrictive housing regulations might hinder such arrangements.

We begin by exploring the patterns of living arrangements around the world and the connection between living arrangements and marital status in Egypt. We illustrate the rising trend in independent living among married couples and thus the linkage between household formation and housing markets. We then review the housing policy landscape in Egypt, including rent control laws and their liberalization in 1996. Our subsequent multivariate analyses take advantage of the change in housing laws to measure the effects of housing policies on the timing of marriage. Our difference-in-difference method relies on local variation in the availability of housing subject to the new rent law and the fact that the availability of such housing is likely to have a greater effect on cohorts of men and women reaching marriageable age after the passage of the 1996 law.

## Background

### Global housing patterns

Before reviewing the relationship between marriage and housing in Egypt, we present some statistics on housing and living arrangements from census data for comparator countries (Minnesota Population Center 2020), in order to place Egypt in a broader international context. As shown in Table 1, Egypt’s share of households renting is high relative to comparator countries, with the possible exceptions of Iran and Turkey. Nevertheless, the share of households renting declined steadily in Egypt from 1986 to 2006, despite the introduction of the new rent law in 1996. Egypt also stands out in terms of the percentage of households with nuclear family living arrangements. Among all the comparator countries, with the exception of Iran, the share of nuclear households is highest in Egypt and rose steadily from 1986 (83 per cent) to 2006 (91 per cent). There is a tendency for this proportion to increase in most countries over time as couples pursue more ‘modern’ living arrangements, but the increase is particularly pronounced in Egypt.

### Marriage and housing decisions in Egypt

Marriage in Egypt increasingly heralds the formation of a new, independent nuclear household, underscoring the importance of access to housing to the ability to marry. Prospective grooms need to be economically ready for marriage by first finding employment after finishing education, preferably a ‘good’ job that is both lucrative and socially prestigious (Dhillon et al. 2009; Assaad et al. 2010; Egel and Salehi-Isfahani 2010; Assaad and Krafft 2014; Gebel and Heyne 2016; Krafft and Assaad 2020). Once they have secured a good job, men are both more attractive marriage partners and more financially able to accumulate the necessary resources to marry. The most important of these economic preconditions to marriage is the ability to secure acceptable housing for the couple (Amin and Al-Bassusi 2004; Dhillon et al. 2009).

The initial acquisition of housing has been estimated to constitute 38 per cent of total marriage costs in Egypt (Salem 2015). Customarily, the acquisition of housing is primarily the responsibility of the groom and his family. If a groom had to cover the entire cost of marriage from his own savings, it would take an average of eight years of wages in Egypt to cover the cost of marriage. The initial housing costs have been estimated as equivalent to approximately three years of the groom’s wages (Assaad and Krafft 2015a).

The need for personal or familial savings, which may take a long time to accumulate, contributes to marriage delays, which are further compounded by rising education levels and protracted transitions to the kind of work that signals economic readiness for marriage among young men (Assaad et al. 2010; Bongaarts et al. 2017; Nilsson 2019; Krafft and Assaad 2020). Being ready requires, among other things, the ability to secure housing arrangements that are deemed acceptable to the bride and her family. In anthropological research, both young people and their parents explained that increasing costs related to the need to set up a separate household were departures from historical patterns and were contributing to delays in marriage (Amin and Al-Bassusi 2004). Men’s responsibilities, especially the cost of housing and finding a job to pay for marriage costs, are the biggest barriers to marriage (El Feki 2013). Premarital negotiations around housing may even include specifics, such as the location of the flat (Hoodfar 1997).

Delays in the transition to marriage also delay the transition to other adult roles—including independent living, socially sanctioned sexual relations, and childbearing—which are largely restricted to marriage according to prevailing norms in the region (Hoodfar 1997; Rashad et al. 2005; Salehi-Isfahani and Dhillon 2008; Dhillon et al. 2009; Egel and Salehi-Isfahani 2010; El Feki 2013). The delays in marriage and the anxiety associated with them have become known in the literature on youth transitions in the Middle East and North Africa region as ‘wait adulthood’ or ‘waithood’ for short (Singerman 2007, Dhillon et al. 2009). Thus, the functioning of the housing market and associated policies plays a crucial role in enabling or constraining the transition to adulthood.

To illustrate the linkage between marriage and living arrangements, we use data from Egypt’s population censuses to examine the percentage of individuals that live with at least one parent, by marital status, urban/rural location, age group, and sex (Table 2). The vast majority of never-married individuals aged 15–34 live with their parents, with that percentage even rising slightly from 1986 to 2006. This pattern holds for men and women and in both urban and rural areas. While the percentage of never married individuals living with their parents is lower for those aged 35–54, this is presumably because there is a higher likelihood that the parents are deceased. Moreover, given the near universality of marriage by age 40 in Egypt (Rashad and Osman 2000; Salem 2015; Krafft and Assaad 2020), there are fewer never married individuals in this age range. A smaller and declining percentage of currently married individuals live with their parents. Among currently married men aged 15–34, the share living with at least one parent declined from 16 per cent in 1986 to 7 per cent in 2006 in urban areas and from 35 per cent to 10 per cent in rural areas. Since the tradition in Egypt is for the bride to move to her in-law’s family if extended household living arrangements are adopted, an even smaller proportion of currently married women live with at least one of their parents. The sum of the percentages of men and women living with their parents indicates the prevalence of extended households in Egyptian society: extended household living is on the decline, involving no more than 8 per cent of couples in Egypt in 2006.

### Housing policy: Rent control and liberalization

One of the reasons that housing contributes to delays in marriage and adulthood is the poor state of housing policy in Egypt. Evidence from a number of less developed countries demonstrates that housing policy can greatly affect the functioning of the housing market. In particular, substantial public ownership of land, poorly enforced property rights, rent control, and lack of financing are important issues globally. Over-regulation is particularly problematic because it tends to limit supply (Malpezzi 1999; Buckley and Kalarickal 2005).

In part as the result of policy challenges, costs of homeownership in Egypt are atypically high, and there are limited opportunities to acquire low-income housing or starter homes. At the same time, credit in the form of a mortgage is not readily available (Sanders 2005; World Bank 2005; Warnock and Warnock 2008; Dhillon et al. 2009). The housing loans available are typically provided by government agencies, and the private market for residential mortgages is generally quite limited (Erbaş and Nothaft 2005; Hassler 2011). Large down payment requirements and short loan periods are also challenges that exacerbate the unaffordability of mortgage finance (Erbaş and Nothaft 2005).

Rental housing may offer a speedier alternative for accessing housing and does not require substantial capital upfront, but rental units are not necessarily available or affordable, particularly prior to the passage of the new rent law. Historically, Egypt’s rental market was heavily regulated (McCall 1988a). Law 52 of 1969 set rent control policies that persisted until 1996, although there were amendments in 1976, 1977, and 1981 (McCall 1988a; Shawkat 2020). The law, summarized in Table 3, initially required annual rents to be fixed in nominal terms at 3 per cent of the land value at the time of construction plus 5 per cent of the construction costs (McCall 1988a). Amendments subsequently increased these percentages and allowed for 7 per cent inflation, but rents were still very low relative to housing investment costs and actual inflation rates (McCall 1988a). The 1969 law gave tenants the right to retain their tenancy along the original contractual terms indefinitely, even through inheritance (McCall 1988a). However, a 2002 decision by the Supreme Constitutional Court limited the transfer of rent control contracts through inheritance to one generation (Assaad and Ramadan 2008).

Rent control limited the return on investment for housing construction in the formal private sector and therefore caused a reduction in the supply of rental housing (McCall 1988b). The costs and stringency of rent control led to landlords demanding large upfront payments of ‘key money’ or ‘advance rent’ to access formal rental contracts (McCall 1988a; Assaad and Ramadan 2008; Shawkat 2020).

The rental landscape substantially changed with rent liberalization in Law 4 of 1996 (Middle East Library for Economic Services 2011; Shawkat 2020). The law applied only to new rental contracts, leaving existing rent control arrangements intact, hence becoming known at the ‘new rent’ law. Table 3 summarizes the new rent law as compared with previous legislation. The new rent law allowed for rental contracts of definite duration rather than indefinite tenancies (Shawkat 2020). It allowed the landlord to increase rent (which was fixed under the old law) on contract renewal (Shawkat 2020). The landlord could also opt not to renew the contract at the end of the contractual period (Assaad and Ramadan 2008; Shawkat 2020). Furthermore, the law made it substantially easier to evict tenants (Shawkat 2020).

Altogether, the new law was a relatively sweeping rent liberalization, albeit one that grandfathered in pre-existing contracts. The rent liberalization substantially changed the incentives to build and invest in new housing. Young people seeking to leave their natal family, marry, and rent their first unit faced a changed market. For example, between 1996 and 2006 the number of rental households increased 30 per cent, from 2.9 million to 3.7 million, driven primarily by the 1.1 million households subject to the new rent law, as households subject to the old rent law declined from 2.8 million to 2.6 million (The Built Environment Observatory 2018).

## Data

### Data sources

In order to analyse the relationship between housing and marriage, we need data on age at marriage, the nature of the housing market in the individual’s place of birth, and the timing of other key transitions in the individual’s life course, such as education and employment. The study primarily uses the 2012 wave of the Egypt Labor Market Panel Survey (ELMPS). The ELMPS 2012 is the third wave of a nationally representative panel survey first fielded in 1998. The subsequent 2006 and 2012 waves tracked households from the previous wave and households that split off between waves, correcting for observable attrition through panel weights. The waves also added a refresher sample of new households. Sampling for both the base wave and refresher was undertaken as a randomized stratified cluster sample (with the sampling strategy incorporated into the weights; see Assaad and Krafft (2013) for more information on the ELMPS.

We analyse data from the combined sample that includes both previous wave and refresher households. Our final working sample of individuals aged 15–54 from the ELMPS 2012 contains 12,704 men and 12,653 women. For information on local housing markets, we also draw on microdata from the 10 per cent public use sample of the 2006 Egyptian Population and Housing Census, available through the Integrated Public Use Microdata Series (IPUMS; Minnesota Population Center 2015). These are the most recent publicly available census data, and while they predate the ELMPS 2012 wave, since we are trying to describe the housing stock prior to marriage, this is an advantage. As well as this benefit of preceding housing data, we prefer to use the ELMPS 2012 wave because its sample is larger, it is more recent, and there is a longer period post housing reform to observe than for earlier waves.

### Outcome

Our primary outcome of interest is age at marriage, which we model and describe in discrete-time terms from the ELMPS 2012 data (see ‘Methods’ section for details).

### Covariates of interest

We are interested in the impact of rent liberalization and the expansion of new rent housing on age at marriage. The 2006 Census in Egypt includes information on both the type of housing (rental or owned) and the rental law under which the housing is rented (old rent vs new rent). We summarize this information at the district level (the second level of administrative geography in Egypt) and merge these district-level summary statistics into the ELMPS 2012 individual-level data. We have survey data and summary housing data for 250 districts in Egypt (out of a total of 347 districts in the 2006 Census).

Our two key covariates from the 2006 Census are: (1) rental housing as a proportion of all housing in the district of birth; and (2) new rent units as a proportion of all rentals in the district of birth. We focus on new rent units among rentals to separate out the effects of the reform from the general availability of rental housing. We standardize the housing market measures to have a mean of zero and standard deviation (SD) of one, in order to be able to discuss relationships in terms of SDs. We also interact these two key covariates with a dummy for cohorts of men born in 1972 or later and for women born in 1977 or later, that is, those who would have been exposed to the rental reform based on the respective median age at marriage for men and women (as detailed in the ‘Results’ section). The main effect for this dummy (which is based on the ELMPS 2012 data) is included in our models as well.

Information on local housing markets can help to identify the effect of housing on the timing of marriage. Local housing market conditions, not individual housing outcomes, are our key covariate of interest, because we are interested in how reforms in the housing market influence marriage timing. To avoid the potential endogeneity of individual housing and migration decisions, and thus the choice of current place of residence, we use information on the housing market from the individual’s place of birth rather than their location of current residence.

### Controls

We use a rich set of characteristics at the family and individual levels from the ELMPS 2012 as controls for factors that might confound the relationship between housing and the timing of marriage. Specifically, we control for the individual’s labour market status, whether they are in education, their own and their parents’ levels of education, father’s employment status and occupation (when the individual was age 15), number of male and female siblings, age, region of birth, and its urban/rural status. We estimate our models separately for men and women.

## Methods

We begin with descriptive analyses of the change in age of marriage across birth cohorts. We then examine the costs of marriage and housing. From there, we proceed to a multivariate analysis of the determinants of marriage timing. To do so, we construct an annualized longitudinal data set for each individual over time using time-varying information on marital, educational, and employment status, obtained from retrospective data in the ELMPS 2012, and link these data to housing characteristics at the place of birth from the 2006 Census. Each individual is followed from age 15 until the time of first marriage or the time of survey if the individual is still unmarried when interviewed.

Because the age at marriage is recorded in whole years and there are a number of tied observations in each year, we estimate discrete-time hazard models rather than continuous-time models. Such models are common for estimating age at marriage and other key demographic events (Schellekens 2017; Grant and Pike 2019; Zang 2019). One advantage of these models is that they account for the right-censoring of age at marriage (since many individuals observed at the time of the survey are not yet married). We denote marrying at a particular age *a* as *T*_*a*_. The outcome of interest is the probability of marrying at a particular age if an individual has not yet married. This can be characterized by the discrete-time hazard function, *h*_*i,a*_ (Jenkins 1995):

(1) $$h_{i,a}=\text{Pr}\left(T_{a}|T_{a}\geq a\right)$$

Hazards are presented in our descriptive statistics. We use a discrete-time proportional hazards model—the complementary log-log model—for the multivariate analysis. Discrete-time survival analysis can be undertaken with complementary log-log or logit models (Jenkins 1995). The complementary log-log model is advantageous in terms of ease of interpretation. Effects of covariates can be presented as hazard ratios relative to a non-parametric baseline hazard, similar to the Cox continuous-time proportional hazards model. Additionally, survival analysis methods allow each individual to have time-varying characteristics (such as educational enrolment), as well as time-invariant characteristics (such as parental education), which can both be related to marriage timing. We structure the data so that an observation is a combination of an individual and a year of age to enable our use of age-varying covariates (e.g. that being enrolled in education will vary with age). Incorporating covariates *X*_*i,a*_, the complementary log-log model can be estimated as (Jenkins 1995):

(2) $$h_{i,a}=1-\text{exp}\left\{-\text{exp}\left[\theta \left(t\right)-\beta X_{i,a}\right]\right\}$$

or

(3) $$\text{log}\left(-\text{log}\left(1-h_{i,a}\right)\right)=\theta \left(a\right)+\beta X_{i,a}$$

where *θ*(*a*) refers to a series of dummy variables identifying time since age 15. When exponentiated, the estimated coefficients, *β*, can be interpreted as hazard ratios. Each hazard ratio then describes the relationship between a one-unit increase in a covariate and the hazard of getting married relative to the baseline hazard, which can be extracted from *θ*(*a*).

In order to identify the impact of the new rent law in Egypt, we specify a difference-in-difference discrete-time hazard model. Difference-in-difference modelling is a quasi-experimental approach. It takes advantage of longitudinal information (in our case, we have information pre- and post the housing reform) to compare those exposed to some ‘treatment’ (in our case, a higher level of new rent housing) pre- and post the actual implementation of the treatment. The model allows for a constant difference in pre-treatment outcomes and assumes that difference would continue in the absence of the treatment (the so-called parallel trends assumption). A difference-in-difference approach is commonly used in the literature to identify the impact of rent control, when it ends or is reformed (Sims 2007; Autor et al. 2012).

In our case, an observation is a person-year (year of age), with the model also including district-of-birth-level controls, subscripted *d*. This difference-in-difference model is specified as follows:

(4) $$\text{log}\left(-\text{log}\left(1-h_{i,a}\right)\right)\;=\alpha_{1}R_{i,d}+\alpha_{2}N_{i,d}+\alpha_{3}M_{i,c}+\alpha_{4}R_{i,d}\times M_{i,c}+\gamma \;N_{i,d}\times M_{i,c}+\theta \left(a\right)+\beta X_{i,a}$$

*R*_*i,d*_ represents local rental units as a percentage of all housing, estimated based on the 2006 Census using the individual’s district of birth, and *N*_*i,d*_ is local new rent housing as a percentage of all rental housing, estimated likewise. By local, we mean in the individual’s district of birth; we use district of birth rather than district of current residence to allay any endogeneity concerns related to migration. The dummy for men born in 1972 or later and women born in 1977 or later—the cohorts whose marriage timing is most likely to have been affected by the new rent law passed in 1996—is shown by *M*_*i,c*_ (the *c* subscript denotes cohort). The impact of the new rent law is given by the coefficient *γ* on the interaction between local new rent housing as a percentage of all rent housing and being born after the cut-off year. This coefficient represents the change in the hazard of marriage for a man born in 1972 or later, or woman born in 1977 or later, who lives in an area with +1 SD higher new rent housing, controlling for the main effects of cohort and housing conditions. Essentially, our difference-in-difference model compares marriage timing for individuals born in districts with a higher share of new rent housing and those born in districts with a lower share of new rent housing (in 2006), across cohorts (e.g. pre- and post-1972 cohorts of men).

## Results

### Descriptive analysis

#### Trends in age at marriage.

**Figure 1:**
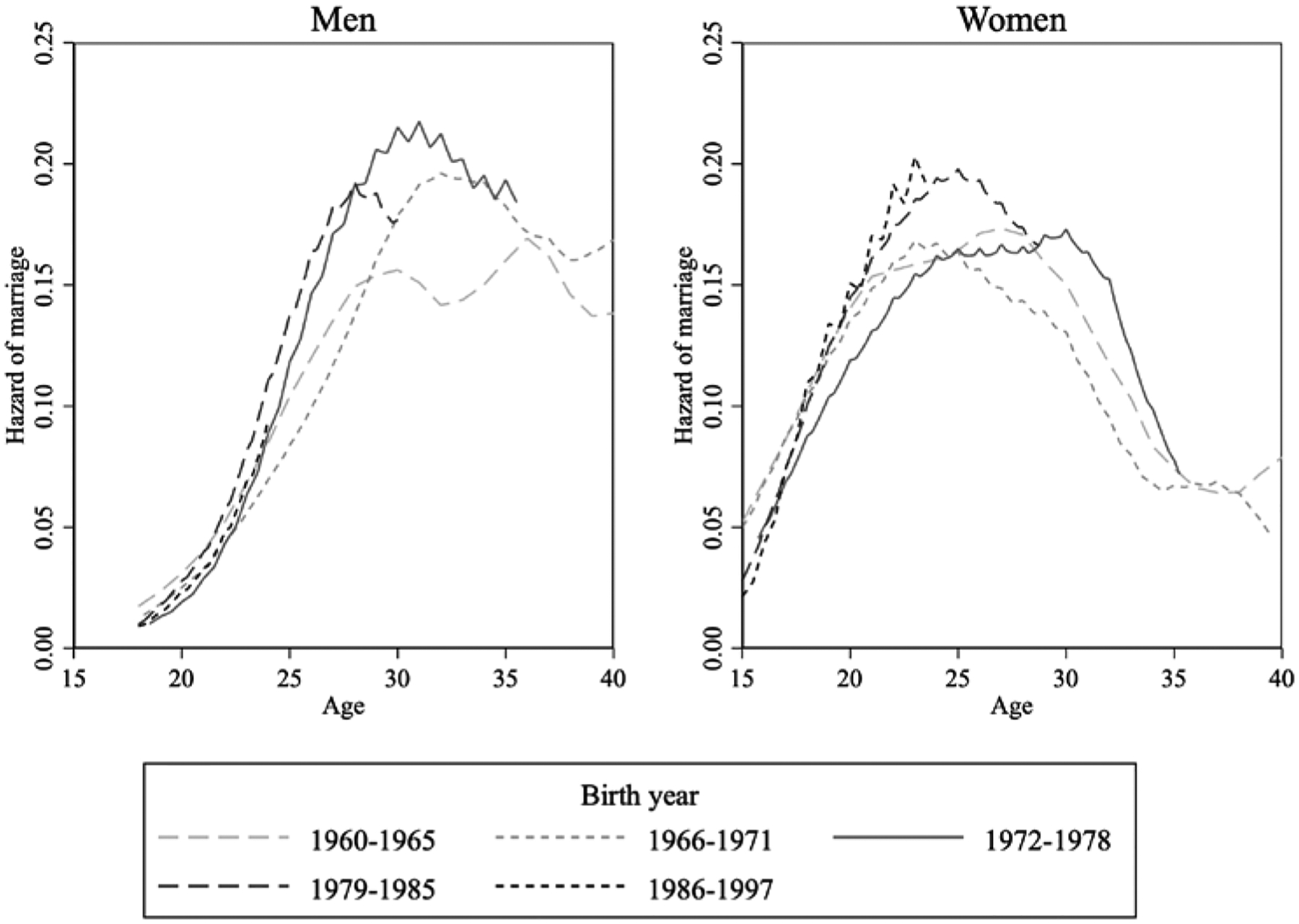
Evolution of the hazard of first marriage by age, birth cohort, and sex: Egypt, 2012 *Notes*: Hazards smoothed with a triangle kernel smoother with bandwidth 4. *Source*: Authors’ calculations based on ELMPS 2012.

We begin our analysis by examining trends in age at marriage in Egypt by birth cohort. Egypt exhibits a striking reversal in the initial increase in marriage age. For men, the median age at marriage (not shown) rose from about 27 for those born in 1960–65 to about 29 for those born in 1966–1971, and then began declining steadily to reach 27 for those born in 1972 and thereafter. Figure 1 shows the evolution of the hazard of first marriage at each age by birth cohort, separately for men and women. The hazards of marriage are much higher around ages 25–30 for those men born post 1972 than pre-1972. A similar pattern is noticeable for Egyptian women. Hazards show rising age at marriage, with hazards shifting to later ages when comparing the 1960–1965, 1966–1971, and 1972–1978 birth cohorts. However, the 1979–1985 and 1986–1997 birth cohorts show a shift back to earlier ages at marriage. In those cohorts the highest hazards are over ages 20–25 (albeit their hazard of early marriage before age 18 is lower than in earlier cohorts).

One of the key hypotheses in this paper is that the reversal of the rising trend in the age at marriage in Egypt can be attributed to developments in the housing market, and in particular to the enactment of the new rent law of 1996. Given that the law applied only at the margin, to new rental contracts, we assume that it took some time for it to show an effect on rental markets, with the earliest effects probably seen no earlier than 1998. Assuming that the average man was marrying at age 27/28, we would expect to begin to see a noticeable impact of the housing law on age at marriage for those born around 1970/71. This period is in fact the point at which we observe the reversal in rising age of marriage in Figure 1. In our empirical tests, we set the cut-off for those who would be affected by the law as men born in 1972 or later to make sure that enough time had passed to allow the law to have an effect on the ground. When the law was passed, the median woman was marrying at the age of 21, meaning that the cohorts of women affected by the law would be those born in 1977 and later. This is in fact the birth cohort where we observe the reversal for women in Egypt.

The reversal in the trend towards later age at marriage, timed around the housing reform, happened despite other factors continuing to trend in a direction that would lead to later age at marriage. Education continued to rise across cohorts and more educated individuals tend to marry later (Assaad and Krafft 2015b; Salem 2015). Adverse developments in the labour market, particularly the decline of public sector employment (Assaad and Krafft 2015c), should have pushed age at marriage higher (Krafft and Assaad 2020). Rising expectations for adult living, including the need to secure independent housing (Singerman 2007; Assaad and Barsoum 2009), would be expected to increase age at marriage further. The housing market, in particular rent liberalization, is the rare factor that would decrease age at marriage. Egypt is relatively unusual in the region for exhibiting a decline in age at marriage. For example, Tunisia has experienced large increases in age at marriage over several cohorts (Assaad et al. 2017). Jordan has experienced more modest increases, but also its rental market is notably more robust than Egypt’s or Tunisia’s (Assaad et al. 2017).

#### Marriage costs and housing.

We now move to an examination of housing affordability ratios and the share of initial housing costs in total marriage costs for newly married couples (in 2006 and 2012), as presented in Table 4. We focus on costs for marriages in the ten years preceding each survey and compare those statistics, due to partial inflation of retrospective costs that confounds the assessment of trends by year of marriage (Assaad et al. 2018). We construct an affordability ratio by dividing the initial cost of housing at marriage by the current annual monthly wages of the groom. Note that these are current annual wages, and thus likely to be higher than wages prior to marriage and so may lead to an underestimate of the affordability ratios. In both 2006 and 2012, Egyptian grooms would have had to devote around two full years of wages (22 months in 2006 and 25 in 2012) to cover their initial housing costs if they were paying them in full. In terms of the groom’s share of the housing costs, they are equal to almost a year (ten months) of his wages after accounting for the fact that the groom’s family helps, as do the bride and her family to a much lesser extent. From examining the share of initial housing costs relative to the total costs of marriage, we confirm that housing costs are a substantial obstacle to marriage in Egypt. It is notable that the share of housing costs in the marriage budget in Egypt in 2012 was 29 per cent. Overall costs and housing costs have risen over time, driven by increasing standards of living and the increase in nuclear family living shown in Table 1.

#### New rent housing in Egypt.

In order to analyse the effects of the new rent law in Egypt, we use data from the ELMPS 2012. First, we calculate the mean cost of housing at marriage for men who identify as renters at the time of marriage and who married in the preceding ten years. We find that the average initial cost of housing at marriage for those renting under the old rent law is approximately 14,777 Egyptian pounds (£E) compared with approximately £E10,772 for those renting under the new rent law. (One US$ was equal to £E6.1 in 2012 [World Bank 2013].) We also calculate the median initial cost of housing at marriage under both laws, in case outliers skew the means. For those renting under the old law, the median is approximately £E10,000, whereas it is £E5,000 under the new rent law. Initial housing costs still present under the new rent law could include making the unit ready for habitation (repairs, painting, utilities start-up), as well as first and last month of rent and security deposits. Although the ELMPS 2012 does not collect data on monthly rent, the 2010–11 Household Income, Expenditure and Consumption Survey does, and shows a mean annual rent of £E3,401 in new rent (uncontrolled) units and £E2,883 for old rent (controlled) units (Attia 2016). Using both measures of central tendency, we find that those who rent under the new rent law incur lower initial housing costs at marriage than those who rented under the old rent law, which most likely involved putting down a large amount upfront in key money or advance rent to obtain a rental contract.

### Multivariate analysis: Timing of marriage and the housing market

We estimate a discrete-time proportional hazards model of age at marriage and use a difference-in-difference specification to test for the effect of the enactment of the new rent law in 1996 on the timing of marriage. Given the prevailing ages at marriage among Egyptian men and women at that time, we expect the law to affect the marriage prospects of the cohorts of men born in 1972 or later and women born in 1977 or later. We therefore argue, as described in the ‘Methods’ section, that the effect of the law is identified by the interaction terms between the dummy for men born in 1972 or later and women born in 1977 or later and the variable capturing the percentage of housing subject to the new rent law among all rentals in the individual’s district of birth in 2006. We also include the interaction between the birth cohort dummy and rental housing as a percentage of all housing. The non-interacted forms of these two variables captures the potential selection effect of being born in districts with high percentages of rental housing and high percentages of new rent housing among rental housing, including any time-invariant confounding variables that are associated with a high prevalence of rentals or new rent units and age of marriage. As a sensitivity analysis, we also estimated a model with district-level fixed effects; the results were substantively similar.

Table 5 displays the results of these estimates in the form of hazard ratios, centred around one, separately for men and women. A hazard ratio greater than one implies that the timing of marriage is accelerated by a one-unit increase in the covariate, while marriage is delayed when the ratio is smaller than one. We find that a one-standard-deviation increase in the percentage of rentals under the new rent law increases the hazard of marriage by approximately 14 per cent for men born in 1972 or later and 12 per cent for women born in 1977 or later. This finding is perhaps the most important result of this paper. This result confirms that the reversal of the trend in men’s age at marriage, first noted by Assaad and Ramadan (2008) based on 2006 data, has been sustained and can be attributed to the introduction of the new rent law, as they suggested. The increased availability of new rent housing over time potentially explains the reversal in the trend towards later ages at marriage, with a shift back towards earlier marriage among exposed cohorts, as observed in Figure 1.

Furthermore, we find that a +1 SD increase in rentals as a percentage of all housing also increases the hazard of marriage by about 10 per cent for men born in 1972 or later and by 9 per cent for women born in 1977 or later. Both results confirm that the new rental law contributed to reversing the earlier rising trend in the age at marriage. The hazard ratio associated with the non-interacted new rent percentage, indicating the pre-1972/1977 cohort effect for men and women, respectively, is barely above (and insignificantly different from) one. The hazard ratio associated with the share of rentals in all housing is also insignificant for men and significant for women but below one, suggesting that, if anything, living in areas with a high proportion of rentals is generally associated with later marriage. Thus, if a selection effect does exist, it appears to be limited and going in the direction of later marriage in high rental areas. No significant effect is associated with the non-interacted dummy for being born in 1972/1977 or later, suggesting that the observed reversal in the age of marriage occurred only for those born in areas with high shares of rentals and high shares of new rent units among rentals.

The effects of other covariates, presented in full in Appendix Table A1, are mostly in the expected direction. The effect of employment status on the timing of marriage, as well as its potential endogeneity, is explored in Krafft and Assaad (2020). Since employment and education could be endogenous, as a sensitivity analysis we reran our model excluding educational level, being in education, and labour market status. The results without labour market status and education were substantively similar.

Employment in the public sector and in the formal private sector tends to speed up marriage for men in Egypt relative to informal private sector employment (the reference category), as it is an effective signal that a man is economically ready for marriage. Unemployed men and those out of the labour force experience a much lower hazard of marriage compared with those in employment, as expected. Women, by contrast, tend to work in private sector informal wage jobs to accumulate savings prior to marriage, then leave such work in anticipation of marriage, such that all other states show significantly higher hazards of marriage; for women there is no penalty to being unemployed or out of the labour force, given traditional male-breadwinner/female-homemaker norms.

As expected, being in education delays marriage considerably, but the effect of education goes beyond the effect of simply being in education. The hazard of marriage falls steadily with the level of education for men, but shows a complex pattern for women. Controlling for own education, the hazard of marriage has a complex relationship with parental education, which is presumably a proxy for social class. Being from a higher social class could contribute to the family’s ability to pay the costs of marriage, but it could also raise expectations about the potential spouse and the quality of the marriage to be achieved (Assaad and Krafft 2015d). Our results suggest a non-linear relationship between social class and the timing of marriage. For instance, among men the hazards of marriage are lowest for those whose mothers have basic education and highest for those whose mothers have higher education.

### Simulations

**Figure 2:**
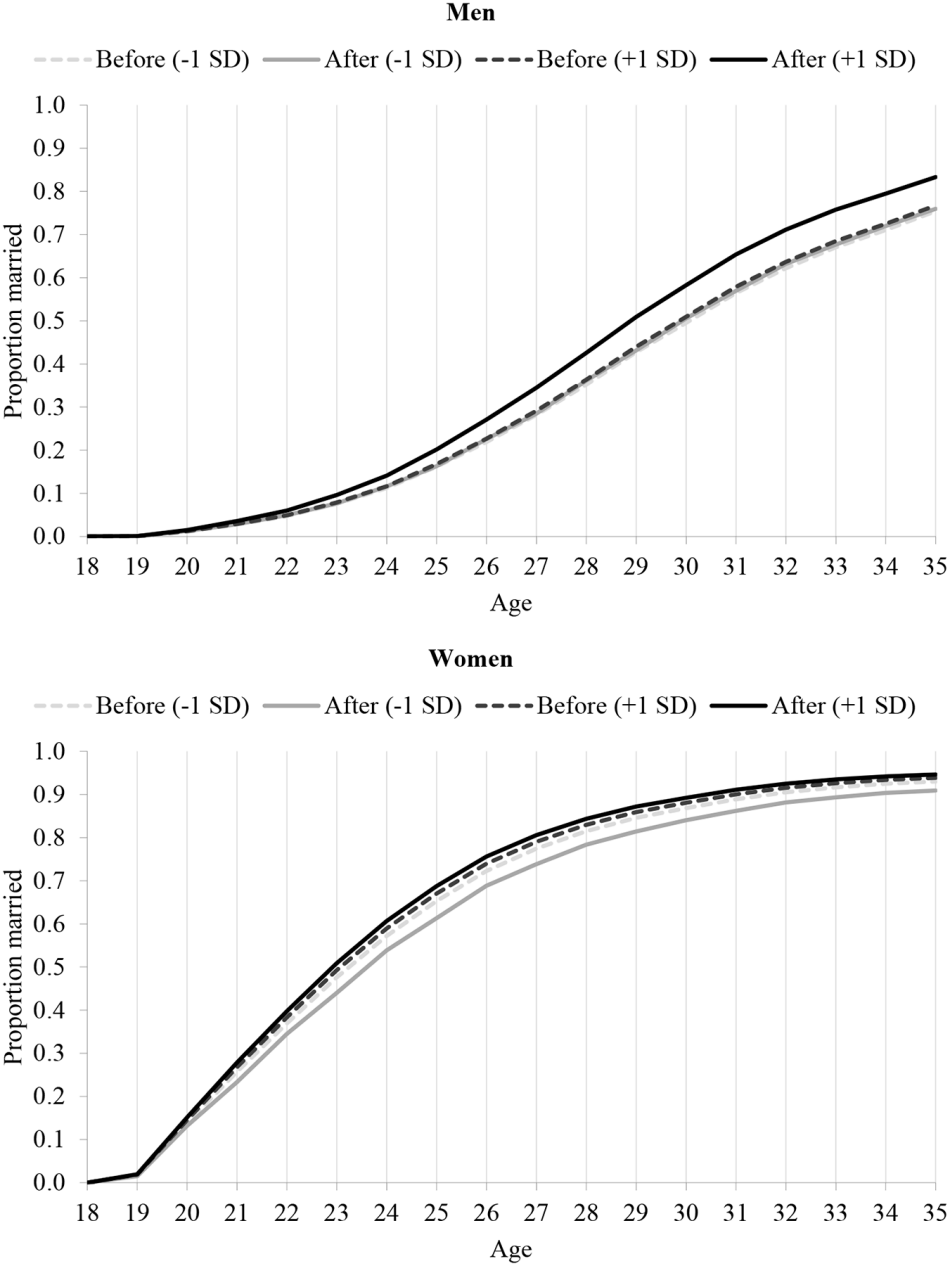
Proportion married at each age simulating variation in new rent units as a share of all rental housing for those born before 1972/77 and in 1972/77 or later, by sex: Egypt, 2012 *Notes*: The four lines on each chart represent cohorts and new rent units as a proportion of all rental units as follows: Before = born before 1972 (men) or 1977 (women); After = born in or after 1972 (men) or 1977 (women); −1 SD = proportion of new rent units in district of birth is one standard deviation below the average; +1 SD = proportion of new rent units in district of birth is one standard deviation above the average. Simulations are conducted for a reference individual with the following characteristics: secondary-level education; in education until age 18; private informal wage worker (if male) or out of the labour force (if female); parents both educated up to secondary level; father professional private sector wage worker when individual was aged 15; born in Greater Cairo; has one brother and one sister; and is 35 years old at survey. These characteristics were selected as a relatively ‘typical’ profile. For instance, ‘private informal wage work’ is the most common employment status for men and ‘out of the labour force’ for women; secondary is the most common education level in Egypt; and Greater Cairo is the most populous region. Age 35 is the midpoint of ages we consider. *Source*: Based on models presented in Table 5.

In order to illuminate the relationship between housing and the timing of marriage further, we use the results of our multivariate analysis to predict what happens to the age of marriage as we change the characteristics of local housing markets. Specifically, we vary housing market characteristics from −1 SD below the mean to +1 SD above the mean and predict the proportion married at each age. The simulations are conducted for a reference individual with secondary-level education, who was in education until age 18 and then was a private informal wage worker (if male) or out of the labour force (if female), whose parents were both educated up to the secondary level, and whose father was a professional private sector wage worker when the individual was aged 15. In addition, the reference individual was born in Greater Cairo, has one brother and one sister, and is 35 years old at the time of the survey. Figure 2 depicts how housing market variables affect the proportion of respondents married at each age, equivalent to a failure function in survival analysis terms.

We simulate the interaction between new rent units as a share of all rental units and the dummy indicating men born in 1972 or later and women born in 1977 or later (Figure 2). A shift of the curves to the left indicates faster transition to marriage. The most notable result is that increasing the proportion of new rent units among rentals by 2 SD (from −1 to +1) shifts the curve substantially to left for males born since 1972. The median age of marriage for this group falls from 30 to 29 if their place of birth shifts from −1 SD below to +1 SD above the mean in terms of the proportion of new rent housing. For those men born prior to 1972, the change is much smaller. For women, because the direction of the main effect of being born in 1977 or after is in the opposite direction, the gap between cohorts born before and after 1977 for the proportion married at +1 SD of new rent housing is less dramatic, but the gap between −1 SD and +1 SD for those born in or after 1977 is quite large and better denotes the effect of the law. Notably, women’s median age at marriage in the simulation is 24 for every status except for those born in or after 1977 in districts where the proportion of new rent units is +1 SD, for whom the median age at marriage is 23. We posit that the shifts in age at marriage can be attributed to the introduction of the 1996 housing law. These simulation results control for rentals as a proportion of all housing. If the new rent law resulted in an increase in the availability of rentals overall, as we would expect, the effect of the law on speeding up marriage would be even larger.

## Conclusion and policy implications

Housing plays an important role in enabling young people in Egypt to marry, a crucial stage in their transition to adulthood, in a context where independent living at marriage is increasingly becoming the norm. Although Egypt shares with other parts of the world a trend towards later marriage for young women, it is distinctive in terms of its substantial increases in age of marriage among young men as well, and, therefore, its fairly persistent and sizeable spousal age gaps (Mensch 2005). While demographers and policymakers generally applaud delaying marriage among young women and reducing teenage marriage rates, there is considerably more social and policy anxiety when men’s age at marriage rises as well, especially in a context where the transition to adulthood is strongly predicated on marriage (Assaad and Barsoum 2009; Salem 2016). The rise in age at first marriage in Egypt is widely attributed to men’s growing inability to signal economic readiness for marriage in a cultural milieu where men and their families are still expected to shoulder the bulk of the economic burden, including provision of the marital dwelling (Assaad et al. 2010; Krafft and Assaad 2020). In such a context, housing policies and the functioning of housing markets play a critical role in facilitating the transition to marriage. When the acquisition of housing requires the accumulation of large sums of money upfront, the economic burden on men increases and marriage will likely be delayed. If, on the other hand, it is possible to acquire rental housing fairly easily or finance purchases with long-term loans, the initial costs of housing will not loom as large.

In light of the analyses presented in this paper, we are able to gain insights into the timing of marriage and functioning of housing markets. We first noted the dramatic reversal in the trend in age at marriage among Egyptians, with a fairly sharp rising trend that reversed for young men born around 1972, and likewise for young women born around 1977. We then noted, as Assaad and Ramadan (2008) had before us, that the cohorts experiencing this reversal were exactly the same as those who would have been affected by the new rent law passed in 1996, which made it easier to acquire market-rate rental housing. We formally tested the effect of the law on the timing of marriage using a difference-in-difference set-up. This approach allowed us to distinguish between the effects of the new law and the effect of time-invariant unobservables that may be correlated with both increased availability of rentals overall and new rent units, and with the timing of marriage.

We found that the introduction of the new rent law did increase the hazard of marriage for young men born in 1972 or later and young women born in 1977 or later. Each 1 SD increase in new rent units as a proportion of rental housing in a young person’s district of birth increased the hazard of marriage, by 14 per cent for men born in 1972 or later and 12 per cent for young women born in 1977 or later compared with those born earlier. The main effect of this variable was small and insignificant, suggesting that selection on unobservables is not a major concern. Similarly, a 1 SD increase in overall rental units as a proportion of all housing raised the hazard of marrying for men born in 1972 or later by 10 per cent (and for women born in 1977 or later by 9 per cent) relative to those born earlier. In contrast, the main effect of this variable, which probably captures the effect of selection, was a reduction of 8–9 per cent in the hazard of marrying (significant for women but not men). This suggests that places with relatively high availability of rentals are associated with other attributes that raise the age of marriage (e.g. urban living, more modern marriage aspirations). It is thus necessary to correct for such selection to detect the effect of a greater availability of rentals on the timing of marriage, which is precisely what we were able to do in this paper.

Our results indicated that rent liberalization decreased initial housing costs and accelerated marriage in Egypt. The previous policy of rent control, although meant to reduce the housing cost burden in Egypt, in fact contributed to rising initial costs of housing and delays in marriage. Rent control dried up the supply of rental housing or made renting contingent on young couples coming up with large upfront sums for key money or advance rent, or for outright housing purchase or construction. Once the rent liberalization policy was implemented to allow for market-rate rentals, age at marriage began falling. Even if they must pay more rent on an ongoing basis (Attia 2016), young people are now at least able to make an earlier start to their marital lives.

Another policy that could have similar effects is to substantially increase the availability of housing finance (Chiuri and Jappelli 2003; Erbaş and Nothaft 2005). However, developing a comprehensive and inclusive housing finance system in conjunction with appropriate government regulation is a long-term task (Renaud 1984; Okpala 1994). It is also challenging to set up such a system in a context characterized by widespread informality of employment, as is the case in Egypt, where recent data indicate that formal employment constituted no more than 38 per cent of total employment in 2018 (Assaad et al. 2019). In the meantime, more flexible rental housing regulations can go a long way in resolving the housing shortages faced by prospective couples.

These results have important implications for global housing policy, as countries throughout the world struggle with the design of housing policies to achieve both economic and social goals (Dübel and Brzeski 2006; Ballesteros et al. 2016). While rent control does decrease prices of rent-controlled units for tenants, it raises prices for uncontrolled units (Early 2000; Roland and Svarer 2002; Diamond et al. 2018; Mense et al. 2018). It also reduces the supply of rental housing (Diamond et al. 2018). Although intended to increase the security and affordability of housing for *existing* tenants, we have demonstrated that such policies appear to have particularly negative effects on the formation of *new* households. This additional—and previously unresearched—impact of rent control should be considered in global housing policy.

## Figures and Tables

**Table 1:** Percentage of households with rented housing and percentage of households with nuclear family living arrangements: Egypt and comparator countries, by year

Country	Year	Percentage of households in rented housing	Percentage of households with nuclear family living arrangements
Egypt	1986	37	83
	1996	27	87
	2006	22	91
Bangladesh	1991	8	61
	2001	10	65
	2011	14	69
Indonesia	1990	8	73
	2000	–	77
	2010	12	75
Iran	2006	22	90
	2011	–	94
Morocco	1994	21	69
	2004	19	71
	2014	19	74
Philippines	1990	9	75
	2000	10	76
	2010	10	73
Turkey	1990	31	77
	2000	24	77

*Note*: ‘–’ denotes not available.

*Source*: Authors’ calculations using population census microdata from IPUMS-International (Minnesota Population Center 2020).

**Table 2:** Percentage of individuals living with own parent(s) by marital status, age group, and urban/rural location: Egypt, 1986, 1996, and 2006

	Never married				Currently married				Previously married		
	1986	1996	2006		1986	1996	2006		1986	1996	2006
Males											
Urban											
15–34	87	92	90		16	11	7		47	57	55
35–54	35	60	55		6	5	4		14	20	18
All	85	91	89		9	7	5		24	28	26
Rural											
15–34	89	95	96		35	22	10		50	62	61
35–54	30	63	61		13	10	6		12	21	15
All	87	95	96		23	14	8		26	36	32
All											
15–34	88	94	93		26	18	9		49	59	58
35–54	33	61	57		9	7	5		13	20	17
All	86	93	93		15	11	7		25	31	29
Females											
Urban											
15–34	90	96	93		3	2	2		55	52	55
35–54	29	59	62		1	1	1		9	9	9
All	88	95	92		2	2	1		18	15	16
Rural											
15–34	87	97	97		3	1	1		59	50	53
35–54	24	55	60		1	0	1		9	7	7
All	84	96	96		2	1	1		19	14	14
All											
15–34	89	96	95		3	2	1		57	51	54
35–54	27	57	61		1	1	1		9	8	8
All	86	95	94		2	1	1		18	15	15

*Source*: As for Table 1.

**Table 3:** Provisions of rental laws in Egypt

	Law 52 of 1969 (and amendments) (old rent)	Law 4 of 1996 (new rent)
*Duration of contracts*	Indefinite	Definite
*Renewal*	Renters have right to remain	Landlords can decide whether or not to offer renewal
*Eviction*	Renters can only be removed under exceptional circumstances, required court proceedings	Tenants can be evicted without court proceedings
*Cost of rent*	1969: fixed annual rents in nominal terms at 3 per cent of the land value at the time of construction plus 5 per cent of the construction costs; 1977: 7 per cent of land values in 1974 and 10 per cent of the construction costs, allowing for 7 per cent inflation; 1981: 7 per cent of land and building cost for units built after 1981 using real costs	Landlords can change the rent at their discretion at the end of the contract period (on renewal)
*Applicability*	Pre-1996: applied to all rentals; Post 1996: still applied to rent contracts signed pre-1996	Applied to new rental contracts (and new buildings) 1996 and later

*Source*: McCall (1988a); Middle East Library for Economic Services (2011); Shawkat (2020).

**Table 4:** Housing and marriage cost ratios for men: Egypt, 2006 and 2012

	Initial housing cost ÷ groom’s monthly wage (in months of wage)	Initial housing cost paid by groom ÷ groom’s monthly wage (in months of wage)	Housing costs ÷ total cost of marriage (percentage)	N (observations)
*2006*	21.8	9.8	23.9	2,989
*2012*	25.2	10.4	28.5	4,591

*Notes*: Restricted to men who married in the preceding ten years. Spousal reports of marriage costs used for 2006, as men were not asked directly in that year.

*Source*: Authors’ calculations using ELMPS 2006, 2012.

**Table 5:** Hazard ratios from discrete-time proportional hazards model for age at marriage, by sex: people aged 15–54, Egypt, 2012

	Men	Women
Men born 1972 or later / women born 1977 or later	1.078	0.939
	(0.060)	(0.043)
Rental housing as a percentage of all housing (SD) × men born 1972 or later / women born 1977 or later	1.104*	1.091*
	(0.055)	(0.042)
New rent housing as a percentage of rental housing (SD) × men born 1972 or later / women born 1977 or later	1.139**	1.116**
	(0.048)	(0.039)
New rent housing as a percentage of rental housing (SD)	1.020	1.024
	(0.037)	(0.034)
Rental housing as a percentage of all housing (SD)	0.914	0.924*
	(0.046)	(0.036)
Controls included	Yes	Yes
Age in year included	Yes	Yes
Rural residency and region dummies included	Yes	Yes
*N* (person-years)	132,717	89,896
*N* (individuals)	12,702	12,653

* p<0.05;

** p<0.01;

*** p<0.001

*Notes*: Standard errors (in parentheses) are clustered at the primary sampling unit (PSU) level. Individual and family characteristics included as controls are the individual’s labour market status, whether the individual is in education, own and parents’ levels of education, father’s employment status and occupation, and number of male and female siblings.

*Source*: Authors’ calculations using ELMPS 2012 and population census microdata from IPUMS-International (Minnesota Population Center 2015).

## Appendix: Table A1

**Table A1:** Hazard ratios for all covariates from discrete-time proportional hazards model for age at marriage, by sex: people aged 15–54, Egypt, 2012

	Men	Women
Men born 1972 or later / women born 1977 or later	1.078	0.939
	(0.060)	(0.043)
Rental housing as a percentage of all housing (SD) × men born 1972 or later / women born 1977 or later	1.104*	1.091*
	(0.055)	(0.042)
New rent housing as a percentage of rental housing (SD) × men born 1972 or later / women born 1977 or later	1.139**	1.116**
	(0.048)	(0.039)
New rent housing as a percentage of rental housing (SD)	1.020	1.024
	(0.037)	(0.034)
Rental housing as a percentage of all housing (SD)	0.914	0.924*
	(0.046)	(0.036)
*Labour market status (ref = private informal wage)*		
Public	1.365***	2.655***
	(0.051)	(0.267)
Private formal wage	1.192***	1.307
	(0.050)	(0.199)
Non-wage	1.107*	2.000***
	(0.045)	(0.288)
Unemployed	0.513***	2.241***
	(0.050)	(0.254)
Outside labour force	0.305***	2.467***
	(0.020)	(0.233)
*In education*	0.685***	0.194***
	(0.050)	(0.009)
*Education (ref = none)*		
Reads and writes	0.879	1.015
	(0.062)	(0.065)
Basic	0.825***	1.211***
	(0.041)	(0.050)
Secondary	0.722***	1.358***
	(0.033)	(0.052)
Post-secondary	0.721***	0.971
	(0.046)	(0.058)
University	0.620***	0.879**
	(0.031)	(0.038)
Postgraduate	0.675***	1.738***
	(0.079)	(0.224)
*Mother’s education (ref = none)*		
Reads and writes	0.930	1.141**
	(0.050)	(0.047)
Basic	0.814**	0.890*
	(0.052)	(0.048)
Secondary	0.927	0.899*
	(0.070)	(0.048)
Higher education	1.035	0.820*
	(0.115)	(0.067)
*Father’s education (ref = none)*		
Reads and writes	0.928*	0.970
	(0.034)	(0.034)
Basic	0.751***	0.835***
	(0.036)	(0.036)
Secondary	0.877	0.895*
	(0.062)	(0.049)
Higher education	0.804**	0.799**
	(0.067)	(0.059)
*Father’s employment status (ref = public)*		
Private wage work	0.948	0.991
	(0.046)	(0.042)
Employer	0.985	0.995
	(0.055)	(0.048)
Unpaid family worker	0.794**	0.803***
	(0.059)	(0.052)
Missing/do not know	1.091	0.800
	(0.278)	(0.230)
*Father’s occupation (ref = manager)*		
Armed forces	1.048	1.331**
	(0.167)	(0.142)
Professionals	0.898	1.075
	(0.080)	(0.076)
Technicians and associate professionals	0.973	1.063
	(0.073)	(0.069)
Clerical support workers	0.995	1.051
	(0.105)	(0.105)
Service and sales workers	0.930	0.899
	(0.077)	(0.060)
Skilled agricultural workers	0.967	0.986
	(0.058)	(0.054)
Craft and related trades workers	0.943	0.926
	(0.072)	(0.057)
Plant and machine operators	0.951	0.968
	(0.068)	(0.072)
Elementary occupations	0.922	1.002
	(0.069)	(0.064)
Number of brothers (living and dead)	0.997	1.033***
	(0.007)	(0.008)
Number of sisters (living and dead)	1.034***	0.999
	(0.009)	(0.006)
64 − Age	0.955**	0.932***
	(0.014)	(0.009)
(64 − Age)^2^ / 100	1.132***	1.150***
	(0.027)	(0.017)
Constant	0.009***	0.075***
	(0.002)	(0.013)
Rural residency and region dummies included	Yes	Yes
*N* (person-years)	132,717	89,896
*N* (individuals)	12,702	12,653

* p<0.05;

** p<0.01;

*** p<0.001

*Note*: Standard errors (in parentheses) are clustered at the PSU level. *Ref* denotes the reference category (omitted variable).

*Source*: As for Table 5.
